# “The PO-Driven Model”: A Basic Science Pipeline for the Bioeconomy with Solutions Inspired by Convergent Evolution for Connecting Parallel Research Ideas

**DOI:** 10.1093/icb/icae156

**Published:** 2024-12-19

**Authors:** Tilottama Roy, Jung-Youn Lee, Tomokazu Kawashima, Grey Monroe, Prosanta Chakrabarty

**Affiliations:** Missouri Western State University, Department of Biology, Saint Joseph, MO 64507, USA; University of Delaware, Delaware Biotechnology Institute, Newark, DE 19711, USA; Department of Plant and Soil Sciences, University of Kentucky, Lexington, KY 40546, USA; Department of Plant Sciences, University of California, Davis, Davis, CA 95616, USA; Louisiana State University, Baton Rouge, LA 70803, USA; American Museum of Natural History, New York, NY 10024, USA; Smithsonian, National Museum of Natural History, Washington, DC 20560, USA

## Abstract

Basic science research, also called “curiosity-driven research,” is fundamental work done with no immediate economic goals but rather a focus on discovery for discovery’s sake. However, basic science research is often needed to seed more applied, economically oriented, research. Both basic and applied research efforts are important aspects of the “bioeconomy,” defined here as the contributions to the overall economy from various biology-related fields spanning everything from museum-based natural history research to agricultural food and material production to healthcare. Here, we propose that more collaborative efforts across federal granting agencies in a venture-capitalist-like “PO-driven model” can help drive applied innovation from collaborations facilitated by program officers (POs). POs from NSF, DOE, DARPA, USDA, NASA, and other federal agencies should seek out parallel and complementary research ideas from grantees and provide funds to build teams of researchers who may otherwise be unaware of one another. Researchers working in different fields may also be unaware that the different organisms they are studying independently may have evolved similar traits (i.e., convergent evolution) that POs may recognize and who can then facilitate novel research avenues connecting those independent researchers (we provide examples of some projects inspired by convergent evolution here). In this top-down approach to research funding, the US bioeconomy will be pouring fuel on the fire of scientific productivity in this country.

## Introduction to bioeconomy

“Bioeconomy” can be used as an umbrella term to describe all the ways in which biological research can contribute (directly or indirectly) to the economy (Gallo 2022). Some bioeconomic models focus on sustainable use of biological renewables and the partnership between academia, industry, and society (Aguilar et al. 2018); others focus on biotech and biomanufacturing, including environmental goals like reduction of plastic waste, biogas capture and reduction of agricultural methane and carbon dioxide, and genomic sequencing of microbes for gene mining, or the promotion of economic security at different levels (White House OSTP). Others focus on the educational, workforce development, and aspects of sustainability activities (Pascoli et al. 2022). Others still, such as Wei et al. (2022), see the bioeconomy as the newest stage of evolutionary economic development for human societies that began with hunting and gathering and progressed through agricultural, industrial, and informational economic stages in past centuries.

It should be noted that different countries and regions have their own ideas of what the bioeconomy means for them. We define the bioeconomy to be inclusive of all the definitions above and use it as a catch-all for all the ways biological resources and research contribute to the economy.

In order to realize the full potential of any bioeconomy, numerous institutions, and stakeholders with specific tasks and diverse interests need to collaborate (aided by free interactions between relevant technical, commercial, and administrative institutions and their respective expertise). While modern communication technologies facilitate long-distance collaboration, communication can always be better facilitated by those with knowledge of broad swaths of a given landscape and across different expertise. In the area of federal grants that fund much of the basic and applied research of the US bioeconomy, the people with the most administrative and technical knowledge of large swaths of research areas are program officers (POs). POs at a single agency can see a portion of the playing field that may be limited to one type of research and not another (e.g., NSF POs may see the field of basic science research but not medical or other applied research), while POs collaborating across agencies together can have a bird’s-eye view of the entire playing field and together can cut redundancy (e.g., overlapping calls for grants) but can also connect parallel or complementary ideas creating new research goals.

One way that basic science might influence applied research is through the discovery of convergent evolution. “Convergent evolution” refers to the independent evolution of a biological function in diverse organisms that originate from different ancestries and evolutionary paths. This occurs because these organisms face similar environmental challenges or constraints, which, through adaptation lead some organisms to evolve successful survival strategies by altering genetic materials through adaptation. Similarly, in the world of research, in particular, academia, scientists face the common challenge of identifying funding sources to support their scientific curiosities. Traditionally, individual principal investigators search for funding opportunities that align with their research goals. However, sometimes important research directions from the viewpoint of the investigator do not fit any government agency’s portfolio well. Under these circumstances, the investigator must change their research to fit the available programs or proceed without funding. Here, we try to answer the question: “Is there a better way that could help PIs (principal investigators) more effectively direct their energy and time, using what nature does in convergent evolution, ultimately leading to better outcomes for the bioeconomy?”

To answer that important question, we look at US-based funding agencies and their often parallel tracks that, in theory, have little overlap; however, if the agencies collaborated to connect some of those different kinds of researchers they work with directly, a pipeline for connecting parallel or complementary research areas could be created. This idea of connecting parallel tracts across federal agencies created the “Catalyzing Across Sectors to Advance the Bioeconomy” (CASA-Bio, released in September 2022) initiative initially fueled by an executive order from the Biden Administration (https://www.casa-bio.net/). Here, we call for an expansion of that initiative that directly draws researchers from different disciplines to work together if POs believe those connections can fuel new research opportunities (a “top-down” approach). While cross-agency grant solicitations exist (e.g., “Ecology and Evolution of Infectious Diseases” from NSF, NIH, and USDA), they rely on independent researchers to find each other in the “ground-up” paradigm that currently exists across US funding agencies. We provide some examples of how this top-down approach can work by highlighting instances of convergent biological evolution as examples of potential projects from disparate fields that can create new research areas facilitated by the “PO-Driven Model” we describe below.

## A call for more collaboration across US granting agencies

The National Science Foundation (NSF) is the premier basic science funding agency in the country, if not the world (Appel 2000, Arora and Gambardella 2005). Other more applied federal funding agencies include the National Institutes of Health (NIH), Department of Energy (DoE), Defense Advanced Research Projects Agency (DARPA), US Department of Agriculture (USDA), among many others. Although there are some opportunities for “cross talk” among agencies, and there are some collaborative grants where funds are pooled to support researchers doing work in both applied and basic science—these grants are rare, as are the number of applicants applying for such funding. POs (a generic term used here for employees at these agencies involved with facilitating awards to grantees) rarely talk across agencies, and to our knowledge never specifically suggest that certain grantees team up to use their joint expertise to tackle a novel problem area. This new problem area may be one where the complementary expertise of the scientists would benefit from this new partnership. Truly only the top-down view of POs can see the entire field, and the more POs working to discuss their grantees and networks of scientists, the greater their field of vision will be. We encourage additional networking opportunities among POs from different agencies as well as the creation of a database where PIs can input their information to be searched and sorted and where links (e.g., working on the same questions from different angles) among these scientists can be discovered. We also think giving POs access to funds to seed new collaborations and make these connections should be seen as an investment for growing the larger bioeconomy.

## NSF should be a catalyst

One way in which the US bioeconomy can be fueled is through what we propose as the “PO-driven model,” where POs from basic science institutions like the NSF discuss the applications or funded grants of PIs with equivalent staff from the NIH, DOE, DARPA, NASA, USDA, and other US-based applied research funding agencies. This PO-driven model can help drive innovation in the United States and connect basic and applied research quickly and efficiently. The newly created Technology, Innovation, and Partnerships directorate of the NSF could be the facilitator of these collaborations and cross-agency grants; another possibility is that the White House Office of Science and Technology Policy (OSTP) facilitate or administer these PO meetings/PI databases as it has done with CASA-Bio discussed above. POs familiar with their scientific fields, and researchers have a bird’s-eye view of these fields and can make connections and provide seed funding for new collaborations and scientific connections much like Vannevar Bush and others envisioned after the end of WWII (Bush 1945). The Manhattan Project and other large-scale projects would not be possible without the connection of basic and applied research facilitated by well-connected leaders (like J. Robert Oppenheimer) who could bring experts across various disciplines together.

## How does convergent evolution provide a platform for inter-agency collaboration?

**Fig. 1:**
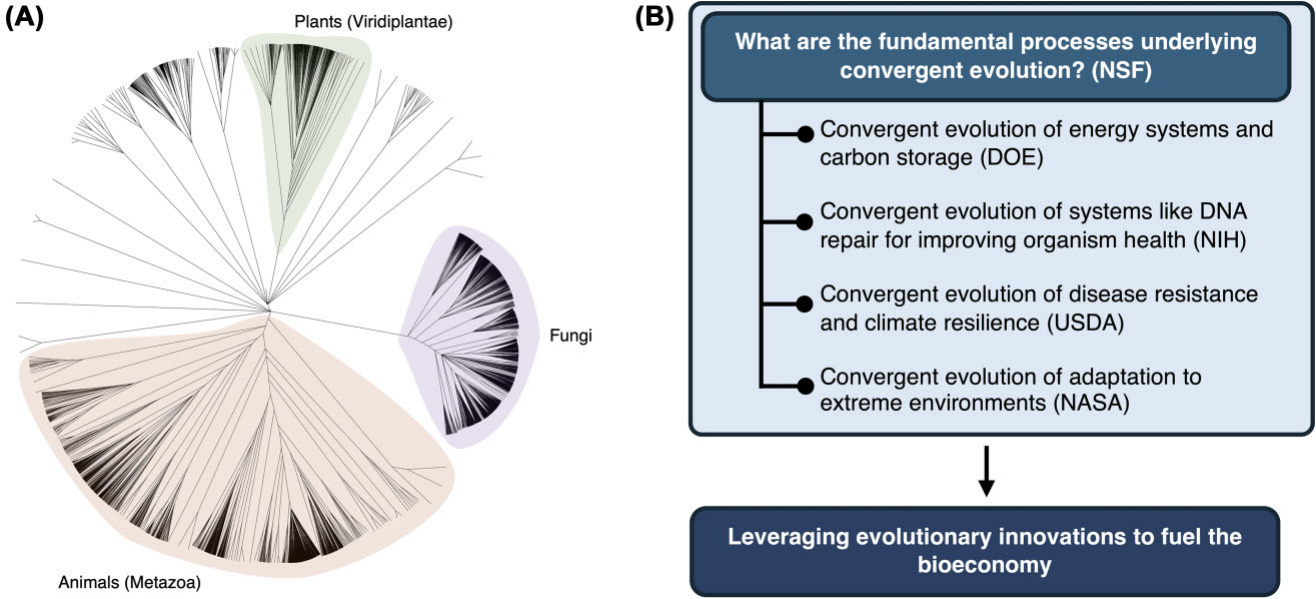
Convergent evolution: a framework for convergence of federal funding of basic and applied research to fuel the bioeconomy. (A) Eukaryotic Tree of Life with whole genome sequences on NCBI (>4000 species). Organisms have been evolving for billions of years to solve many of the same problems we face as a society. We are poised to unlock these innovations through the study of convergent evolution. (B) Examples of linking basic and applied with inter-agency programs. Understanding convergent evolution is a fundamental research goal that not only bridges programs within NSF but also provides a platform for united research agencies.

One area of research that appears to be a natural fit for the PO-driven model is convergent evolution (because convergent solutions in nature may not be known to biological researchers working independently on different organisms, but a group of POs might note these convergences when discussing projects with similar themes). Convergent evolution is a hallmark of natural selection, illustrating how different species independently develop similar traits to overcome similar environments or challenges. Elucidating the underlying processes of convergent evolution, such as selection and mutation, is a key basic science goal and fundamental to understanding the diversity of life (Stern 2013). Uncovering instances of convergence can also provide opportunities for discovering solutions to fuel the bioeconomy and can be noted by POs or PIs who discover convergent solutions in nature being studied in similar or complementary ways by independent researchers. Similarly, POs could identify these noted convergences from basic science projects and work with applied agencies to create collaborative funding to create bridges from basic to applied solutions (Fig. 1). Researchers may be studying evolutionary innovations directly relevant to society—which can be anything from human health, to carbon-based energy storage, to extreme environment adaptation. Advances like whole genome sequencing are opening up the potential to tap into this diversity at Tree of Life scales, toward both fundamental and applied research outcomes.

The NSF is uniquely positioned to facilitate interdisciplinary collaboration among researchers investigating fundamental questions about convergent evolution and those seeking to apply these insights to practical challenges. By partnering with agencies like the NIH, the DoE, the United States Department of Agriculture, and others, the NSF can support a comprehensive approach to leveraging evolutionary knowledge for societal benefit. This collaborative effort aims to harness the power of evolutionary biology to develop innovative solutions for pressing global issues, from improving human health outcomes to advancing sustainable energy technologies and enhancing our ability to adapt to climate change.

Connecting basic science to applied research requires integrating theoretical knowledge with practical applications to solving real-world problems. Providing federal granting agencies a mechanism to foster research collaborations that might not otherwise happen will create a more dynamic and effective research network that better prepares American science for real-world challenges and opportunities. Connecting basic science researchers on parallel science tracts to work on applied applications may ultimately lead to greater economic gains (Bentley et al. 2015) and ultimately an improved bioeconomy (through the creation of new research avenues formed by these novel research collaborations). The following are examples of projects on convergent evolution that could be funded by collaborations between US funding agencies and that were notably formed by the authors of this paper (who are from disparate fields) when NSF POs connected us to discuss convergent evolution (see the “Acknowledgments” section).

### Example 1: convergent evolution project (USDA and NSF): *Arabidopsis* and *Drosophila* and carbon sequestration

The endosperm is a product of double fertilization in flowering plants, developing inside the seed to support embryo development in dicotyledonous (dicot) plants such as peas and beans, and germination and seedling growth in monocotyledonous (monocot) plants such as rice and maize (Shin et al. 2021). The early stage of endosperm development in many flowering plants exhibits a remarkable and unique process, rapid nuclear divisions without cytokinesis (coenocyte). This developmental pattern is strikingly similar to the early embryonic development of the fruit fly *Drosophila melanogaster*, forming a syncytial blastoderm, technically a coenocyte (McCartney and Dudin 2023). This multinucleate development supports the efficient distribution of nutrients and rapid, synchronous nuclear divisions for the swift enlargement of the embryo. Additionally, morphogen gradient formation within the syncytial blastoderm guides subsequent cell type differentiation during embryogenesis.

**Fig. 2:**
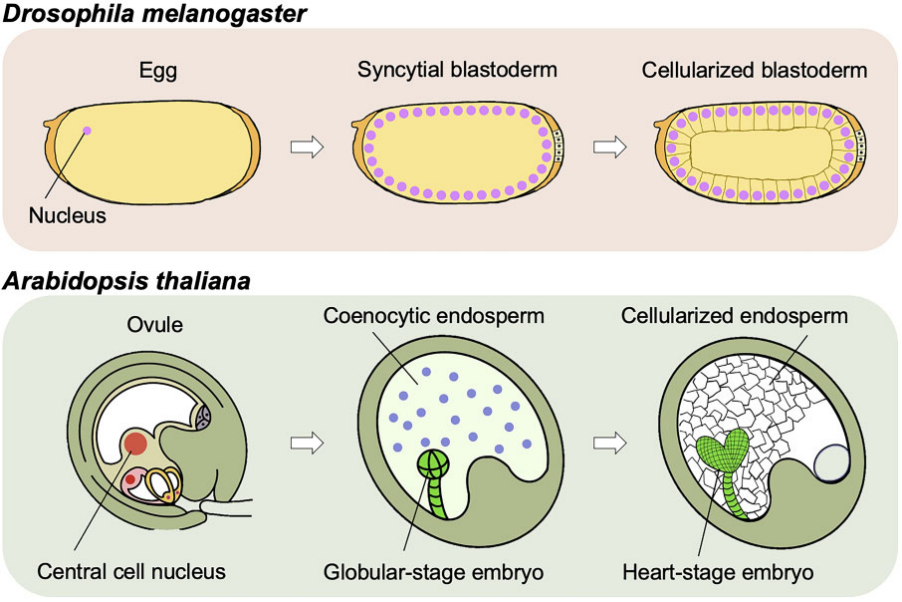
Syncytial/coenocytic development in *Drosophila* and *Arabidopsis. Drosophila melanogaster* embryo develops a syncytial blastoderm, technically a coenocyte, for efficient distribution of nutrients and rapid, synchronous nuclear divisions for swift enlargement. Fertilization of the central cell in many flowering plants, including *A. thaliana*, also triggers nuclear divisions without cytokinesis, generating a coenocytic endosperm. The size and developmental duration of the coenocytic endosperm are associates with the final seed size.

Coenocytic endosperm development in flowering plants precedes embryo development, with rapid nuclear divisions starting just hours after fertilization (Gooh et al. 2015; Ali et al. 2023). As nuclei divide, the endosperm enlarges, and the size of the coenocytic endosperm plays a crucial role in determining the final seed size (Sharma et al. 2024). Similar to the *Drosophila* syncytial blastoderm, the coenocytic endosperm of the model plant *Arabidopsis thaliana* also displays differential gene expression profiles among its subdomains, and both eventually undergo cellularization (Belmonte et al. 2013). This phenomenon exemplifies convergent evolution, where distinct evolutionary paths have led to analogous developmental strategies in both plants and insects, highlighting the effectiveness of this approach in diverse biological contexts, in this case, rapid enlargement (Fig. 2).

Following the characterization of cytoskeleton dynamics controlling the syncytial blastoderm development in *Drosophila*, recent research has revealed the dynamics of the cytoskeleton in the coenocytic endosperm of *Arabidopsis*, highlighting similarities and differences in nuclear positioning mechanisms (Shin et al. 2022; McCartney and Dudin 2023). Manipulating cytoskeleton dynamics in the coenocytic endosperm can alter the final seed size (Ali et al. 2023). However, the exact mechanisms by which cytoskeleton dynamics influence seed size remain unclear. By leveraging knowledge from *Drosophila* research, scientists can better investigate and manipulate the corresponding mechanisms in plant systems, potentially leading to enhanced agricultural outcomes.

In monocot plants, the endosperm serves as the primary food reserve within mature seeds (Olsen 2004). This tissue is rich in starches, proteins, and lipids, providing essential nutrients for the developing seedling upon germination. Beyond its biological role, the endosperm has profound implications for agriculture and the bioeconomy. Enhancing seed size through genetic and agronomic innovations can lead to increased yields, ensuring a more abundant food supply. This is particularly vital for staple crops like rice, wheat, and corn, which are central to global food security. Thus, optimizing endosperm development is a key objective in plant breeding and biotechnology, aiming to meet the demands of a growing population.

Moreover, the seed’s ability to sequester carbon makes it valuable in strategies to mitigate climate change. Seeds represent a stable form of carbon storage, trapping atmospheric carbon dioxide in a durable and long-lived form. By promoting seed production and increasing seed biomass, followed by harvesting and storing seeds, we not only help sequester carbon but also ensure a sustainable food supply when needed. This dual role of the endosperm—supporting both agricultural productivity and environmental sustainability, highlights its potential for innovations that benefit both food systems and the planet.

### Example 2: convergent evolution project (NIH and NSF): cell-to-cell communication and the development of therapeutics

Well-coordinated communication at the level of basic building blocks of an organism is fundamental to its survival. Animal and plant lineages have convergently evolved similar strategies of directly communicating between cells despite their stark differences in body plan and cellular and organismal mobility. This mechanism of direct cell-cell communication involves “tunneling nanotubes” in animals and “plasmodesmata” in plants, respectively. Both types of communication channels are basically cell membrane-lined cytoplasmic bridges that connect neighboring cells to facilitate transport of nutrient, hormone, ions, and signaling or information molecules, including RNAs and proteins.

Plasmodesmata were traditionally considered unique to the plant lineage, making it surprising when tunneling nanotubes were discovered to perform similar roles in animal cells. These intercellular bridges facilitate transmission of not only pro-health signals but also pro-death signals, which play critical roles in immune responses against microbial pathogens. For example, plasmodesmata facilitate movement of photosynthates, ions, and hormones that modulate growth and development of new tissues and organs, while microbial pathogens could mobilize their infectious materials between host cells to spread the infection. Similarly, tunneling nanotubes are implicated in transmitting various signals and even organelles to influence developmental processes and tissue regeneration as well as immune signals determining neighboring cells’ survival or death, and infectious agents like HIV and prions. Understanding the mechanisms of these independently evolved cell–cell communication pathways could lead to the development of innovative applications, such as the design of synthetic cells and tissues, environmentally friendly and human-safe biocides that boost crop yields, and novel therapeutics that intervene or stimulate intercellular connectivity.

### Example 3: convergent evolution and ethnobotany: (NSF, NIH, and USDA)

The chemical similarity of plants belonging to different families that are distantly related, is often considered as a chemosystematic convergence, and can be further developed for commercialization and societal benefit. This convergent evolution of chemicals in plants can similarly be used convergently by different cultures independently for their therapeutic applications due to their similar bioactivity. Phylogenetically informed bioprospecting of medicinal plants relies on interdisciplinary approaches that integrate plant phylogenies, cultural phylogenies, and ethnobotanical data (Garnatje et al. 2017). Funding for such ethnobotanical projects can be supported by a joint initiative formed by a close alliance between funding agencies such as NSF, NIH, and USDA.

## Conclusions

*Why have a birds-eye view if you can't play the puppet master?*

It seems one of the keys to harnessing convergent evolution is to connect funding mechanisms currently working in parallel that support or bring these independent research efforts together. Would not the outcomes be transformative if funding agencies fostered new collaborations and cross-disciplinary research to accelerate the discovery process and facilitate the translation of basic science findings into practical applications?

The convergent evolution examples we provide are just a sample of how connecting researchers from different fields of biology (who were previously unaware of each other) may discover that there are similarities in their study organisms. Although some limited cross-agency federal programs exist to foster, bottom-up, PI-driven approaches, we think additional novel research avenues can be facilitated by the top-down, PO-driven approach we expound here.

## Data Availability

No new data were generated or analyzed in support of this research.
